# Alcohol Marketing during the UEFA EURO 2016 Football Tournament: A Frequency Analysis

**DOI:** 10.3390/ijerph14070704

**Published:** 2017-06-29

**Authors:** Richard I. Purves, Nathan Critchlow, Martine Stead, Jean Adams, Katherine Brown

**Affiliations:** 1 Institute for Social Marketing and UK Centre for Tobacco & Alcohol Studies, Faculty of Health Sciences & Sport, University of Stirling, Scotland FK94LA, UK; nathan.critchlow@stir.ac.uk (N.C.); martine.stead@stir.ac.uk (M.S.); 2 MRC Epidemiology Unit, University of Cambridge School of Clinical Medicine, Cambridge Biomedical Campus, Cambridge CB20QQ, UK; jma79@medschl.cam.ac.uk; 3 The Institute of Alcohol Studies, Alliance House, London SW1H 0QS, UK; KBrown@ias.org.uk

**Keywords:** alcohol marketing, alcohol policy, marketing regulations, sponsorship, frequency analysis

## Abstract

This study examined the frequency and nature of alcohol marketing references in broadcasts of the 2016 UEFA (Union of European Football Associations) European Championships football tournament in the United Kingdom (UK). Eighteen matches from across the tournament were recorded in full as broadcast in the UK, including all four matches featuring the English national team and all seven featuring the French national team. All visual and verbal references to alcohol marketing were recorded using a tool with high inter-rater reliability. A total of 2213 alcohol marketing references were recorded, an average of 122.94 per broadcast and 0.65 per broadcast minute (0.52 per minute in-play and 0.80 per minute out-of-play). Almost all references were visual (97.5%), with 77.9% occurring around the pitch border. Almost all (90.6%) were indirect references to alcohol brands (e.g., references to well-known slogans), compared to only 9.4% direct references to brands (e.g., brand names). The frequency of references to alcohol marketing was high. Although the overall proportion of direct brand references was low, the high proportion of indirect references demonstrates that alcohol producers were able to circumvent the French national law governing alcohol marketing (the Loi Évin) using indirect “alibi marketing”. To ensure the spirit of the Loi Évin regulations are achieved, stricter enforcement may be required to limit exposure to alcohol marketing, particularly for young people.

## 1. Introduction

Sponsorship of high-profile sporting and cultural events is an important and lucrative alcohol marketing strategy. It creates high levels of awareness and allows brands to be associated with attractive and emotionally arousing cultural phenomena such as music or sport [1,2]. Having positive emotional responses to alcohol brands have been associated with increased likelihood of consumption [3,4,5] and alcohol companies have reported increased sales as a result of sponsorship [1]. Exposure to alcohol marketing, including alcohol sports sponsorship, is associated with increased rates of underage and risky drinking [6,7,8]. As well as raising awareness, sponsorship creates positive emotional associations with products and helps to shape norms [2,9]. Consequently, many countries restrict alcohol advertising and sponsorship activities in attempts to protect young people and promote public health [10]. In France, for example, the Loi Évin restricts the placement of alcohol advertising to print media with a largely adult readership (e.g., magazines about travel or home design), the content of which must be based on factual and informative data about the product. No lifestyle messages can be featured and all advertisements must display a mandatory health warning [11,12]. Broadcast advertising of alcohol products on television and radio are prohibited in France, as is alcohol sponsorship of sporting and cultural events [13].

International football tournaments provide a particularly high-profile platform for sponsorship and marketing. The final of the 2014 FIFA (Fédération Internationale de Football Association) World Cup, for example, attracted an average global television audience of around 570 million [14]. Similarly, televised broadcasts of the 2012 UEFA (Union of European Football Associations) European football tournament (typically referred to as the EURO) attracted more than 300 million viewers across 230 worldwide territories [15]. Although the methods used to record the presence of alcohol marketing have differed between studies, previous research has consistently found a high volume of alcohol marketing references present in broadcasts of both tournaments. For example, a frequency analysis of the UEFA EURO 2012 football tournament counted every time a brand logo appeared and found an average of 221.75 references per broadcast [16], whereas a frequency analysis of the 2014 World Cup in Brazil found that alcohol references appeared on screen approximately 100 times per broadcast [17].

The UEFA EURO 2016 tournament differs from previous tournaments in two ways. First, the number of teams featuring at the tournament increased from 16 to 24, which increased the number of matches played and the potential interest from television audiences in competing nations. Second, it was the first time that the tournament had been held in France since the introduction of the Loi Évin in 1991 (France last hosted the Euro tournament in 1984). Despite the legal restrictions on alcohol sponsorship, however, beer producer Carlsberg was again named as one of the 10 key sponsors of EURO 2016 [18]. Carlsberg has sponsored the UEFA EURO tournament since 1988 and claims that this is a profitable relationship, reporting that its sponsorship of the UEFA EURO 2012 tournament “surpassed all expectations” in terms of fan engagement and beer sales [19]. 

Recent research has raised questions about the Loi Évin, with cross-sectional surveys reporting that young people in France recall exposure to alcohol marketing through a range of channels, including at sporting and cultural events, despite such marketing being supposedly prohibited [11]. These findings have wider implications, given that the Loi Évin is often cited as an exemplar of marketing policy by those who advocate for tighter restrictions [20,21,22]. Building on this debate, this study explores the frequency and nature of alcohol marketing references in UK broadcasts of the UEFA EURO 2016 football tournament. By doing so it explores what impact, if any, the Loi Évin had on the presence of alcohol marketing at the tournament and the types of marketing messages that audiences were exposed to during the tournament.

## 2. Materials and Methods

### 2.1. Design

A frequency analysis of all verbal and visual references to alcohol marketing was conducted on 18 matches from the UEFA EURO 2016 football tournament, as broadcast in the UK. The design and approach was informed by previous studies into alcohol sponsorship of televised top class English club football [23] and the UEFA EURO 2012 football tournament [16].

### 2.2. Selection of Broadcasts

Eighteen matches from across the UEFA EURO football tournament were recorded as broadcast in the UK on the public service broadcaster BBC and the main commercial broadcaster ITV (STV in Scotland) (Table 1). Given the UK context of the study, all the matches featuring the English national team were purposively sampled (*n* = 4). As this study considered the effectiveness of Loi Évin, all broadcasts involving the host nation France (*n* = 7) were also purposively sampled to provide insight into matches where international interest and television audiences were likely to be high. This has proven to be the case in previous tournaments where matches featuring the host nation have tended to attract the most viewers globally [14]. All broadcasts involving the Republic of Ireland (*n* = 4) were also purposively sampled, as the introduction of tighter restrictions on alcohol sponsorship, and the implications these may have for hosting international sporting events, were being debated in Ireland at the same time as the tournament [24,25]. As both England and the Republic of Ireland were eliminated from the tournament in the round of 16, two additional matches were randomly selected for the quarter finals, while both semi-finals were recorded. As the final was shown on both ITV and BBC, both broadcasts were recorded. The BBC broadcast was chosen for analysis, as it had the higher audience share [26].

All the selected broadcasts were recorded in their entirety using recordable DVD players. Each recording included normal playing time, added time, extra time, penalty-shoot outs, pre-and post-match interviews and discussion, half-time analysis, and any commercial breaks. The recordings excluded any pre- or post-match discussion, interviews or highlights that were not part of the main scheduled broadcast (e.g., content on on-demand television, content uploaded to sports news sites, and content accessible through interactive television).

### 2.3. Defining Alcohol Marketing References

A reference was defined as any visual and/or verbal reference to an alcohol brand, lasting one second or more, during the broadcasted programme or commercial breaks. In order to capture total exposure, a reference was counted each time it appeared or was heard, irrespective of whether it had been previously seen (e.g., a pitch side advertising board seen in-play first and then again in a replay of that action). A new reference was counted each time the camera changed shot, even if the reference source remained the same (e.g., pitch side advertising first viewed from behind the goal and then again when the shot reverted to the wide side-line angle). A new reference was also counted if a source went out of shot for more than a second (e.g., if the camera panned away from the pitch side advertising and then back again). If multiple different references were presented at the same time (e.g., static and electronic pitch advertising), each was recorded as a separate reference, not combined. If multiple identical references were visible at the same time (for example, if the same brand name or slogan appeared multiple times on the pitch border or on an interview board), they were recorded as “identical references visible at the same time”. 

### 2.4. Codebook Variables

All alcohol marketing references were captured using a codebook that was developed based on two similar previous studies which recorded alcohol references in professional televised football [16,23]. Each reference was coded on the following nominal or continuous criteria. Full definitions are provided in Appendix A.

- Broadcast segment (e.g., pre-match, first half, and half time).
- Location (e.g., pitch border, interview area, and pre-recorded video segments).
- Format (e.g., static advertising, electronic advertising, and commercial advertisement).
- Duration of reference (in seconds).
- Identical reference visible at same time (e.g., multiple pitch borders).
- Alcohol brand featured (e.g., Carlsberg and Fosters).
- Nature of brand reference (e.g., direct reference—such as brand names/logo—or indirect reference—although a name/logo did not appear, the brand was identifiable from other signifiers such as phrases from brand slogans, colours, and typefaces).

### 2.5. Procedure and Inter-Rater Reliability

An initial codebook was developed and piloted on a recorded match from the FIFA World Cup 2014, and revised based on discussion between RP, NC and MS. After being recorded, all 18 broadcasts were systematically coded by RP or NC, while all commercial breaks were coded by an additional researcher (LR). All DVDs were played on Windows Media Player, using the pause and rewind function as required. Data were coded into a Microsoft Excel spreadsheet designed around the codebook. A separate spreadsheet was used for each broadcast. 

RP and NC both coded one broadcast in full (France vs. Germany). Inter-rater reliability was established through percentage agreement on the number of references coded for the categorical variables (e.g., number of references in the pre-match). When averaged for each section of the codebook, there was high agreement for broadcast segment (95%), reference type (98%), reference location (98%), reference format (94%), content of the reference (99%), and which brand was featured (99%). These estimates exceed the suggested 70% threshold for acceptable inter-rater agreement using the percentage measure [27]. A breakdown of agreement by variable is reported in Appendix B.

### 2.6. Ethics

As this study was based on publicly available television broadcasts, and did not include any research participants, no ethical approval was required. 

### 2.7. Data Analysis

Data were analysed using SPSS version 23 (SPSS Inc., Chicago, IL, USA). Frequencies were computed for broadcast segment, location of the reference, format of the reference, brand referenced and content of the reference. Medians and modes were computed for duration of references and number of identical references, due to variation in the distribution of data for these variables. Means were computed for the total number of references per broadcast and the average number of references per broadcast minute (overall, in-play and out-of-play). A series of independent samples *t*-tests were conducted to explore whether the average number of references per match differed by whether the match involved England or not, whether it was broadcast on a commercial channel or not, whether it was broadcast at the weekend or on a weekday, or whether it was broadcast in the afternoon or evening. The average number of references per broadcast minute was computed by dividing the total number of references by the length of each broadcast, and then by dividing the number of references in-play and out-of-play by the respective length of each section in the broadcast. A paired samples *t*-test, based on 2000 bootstrapped comparisons, was used to compare the average number of references per in-play minute to the average number of references per out-of-play minute. Cross-tabulations were used to compare the content of marketing (e.g., direct reference versus indirect reference—see Appendix A) to the location of marketing and the brand that was featured.

## 3. Results

### 3.1. Number of Alcohol Marketing References by Broadcast Characteristic

A total of 2213 alcohol marketing references were recorded across the 18 broadcasts, with an average of 122.94 per broadcast (SD = 42.87) (Table 2). A series of independent samples t-tests found the average number of references per broadcast did not differ dependent on whether a match involved the English national team or not (*p* = 0.64), whether it was broadcast by a commercial (ITV/STV) or a non-commercial broadcaster (*p* = 0.58), whether the broadcast was midweek or at the weekend (*p* = 0.76), or whether the match kicked off in the afternoon or in the evening (*p* = 0.94) (Table 2). 

### 3.2. Alcohol Marketing References per Minute

The 18 broadcasts provided a total of 55.6 h of footage. The average number of references per minute across the 18 broadcasts was 0.65 (SD = 0.17; range: 0.31–0.87). A paired samples *t*-test showed a significant decrease in the average number of references per minute in out-of-play sections (M = 0.52, SD = 0.12) compared to the average number of references per minute during in-play sections (M = 0.80, SD = 0.29), t (17) = −4.43, *p* < 0.001, d = 2.15. The mean difference between out-of-play and in-play sections was −0.28, with a bootstrapped 95% confidence interval of −0.41 to −0.17.

### 3.3. Broadcast Segment

Most references appeared in the first (25.6%) or second halves of standard regulation time (31.6%), followed by the pre-match build-up (16.9%), post-match analysis (11.3%), and half-time discussion (7.5%) (Table 3). Only a small proportion of references were recorded in extra-time (3.9%) and breaks in extra-time (1.2%), as these periods only featured in two of the 18 matches, while penalties only featured in one match (0.3%). Only a small proportion of references featured in commercial breaks (1.7%), and these periods only occurred in the 11 matches broadcast on ITV/STV (BBC programmes do not include commercial breaks). More references appeared during in-play sections (61.5%) compared to out-of-play sections (38.5%). 

### 3.4. Location in Broadcast

Most alcohol marketing references appeared around the pitch border of the featured match (77.9%) (Table 3). The second most frequent location was in pre-recorded video segments (14.7%), which included highlights from other matches from the UEFA EURO 2016 tournament, matches from the qualification stages and matches from other tournaments (e.g., FIFA World Cups and previous UEFA EURO tournaments). Pitch border advertising was also a common feature in these other matches.

### 3.5. Format of Alcohol Marketing References

Almost all references were visual (97.5%). Only a small number were both verbal and visual (2.5%), all of which were sponsorship lead-ins or commercial breaks on ITV/STV. Most references were coded as “Electronic pitch side (all)”, (37.9%); “Electronic pitch side (part)”, (36.0%); or “static advertising” (20.1%) (Table 4).

### 3.6. Duration of Alcohol Marketing References

The median duration of references was 4.47 s (interquartile range = 2.24–9.45). The mode duration was 2 s.

### 3.7. Number of Identical Instances Visible for Each Alcohol Marketing Reference

The median number of identical instances visible alongside each marketing reference was 1.83 (interquartile range = 1.02–5.17). The mode number of identical instances was 1.

### 3.8. Content of Alcohol Marketing References and Brands Featured

Only 9.4% of the references were defined as a direct reference to a brand (i.e., brand name or logo) (Table 4). Nearly two thirds of these direct references were for Carlsberg (60.9%), with the remainder referring to over 26 other alcohol brands (Appendix C). Most direct references appeared in pre-recorded video segments (49.3%), followed by references in the crowd (23.2%) and commercial breaks (17.9%).

Most references were instead indirect brand references (90.6%). Almost all references recorded as indirect were for Carlsberg (99.9%) and comprised either the single word “Probably” or the phrase “…the best in the world”. In both cases, the text appeared in white on a dark green background, the colours associated with Carlsberg, and in the same or a very similar font to that used on their brand logo. Most indirect references appeared on the pitch side border (85.8%) or in pre-recorded video segments (including highlights of other matches) (11.2%).

## 4. Discussion

This study found a high volume of alcohol marketing in UK broadcasts of the UEFA EURO 2016 football tournament, with an average of 122 references per broadcast and average of 0.65 per broadcast minute. This is consistent with previous research that has suggested that alcohol marketing does feature frequently in broadcasts of international football tournaments [16,23]. This study builds on previous research by providing greater insight into the design of marketing, how and where it appeared (e.g., duration, location and the number of identical references), and the nature of the marketing message (e.g., whether the reference to a brand was direct or indirect). 

There was no difference in the number of alcohol marketing references across different broadcasts, including on weekdays versus weekends or matches involving England compared to those involving other teams. Notably, the volume of alcohol marketing was consistent regardless of whether the match was broadcast on a channel that does not carry advertising or sponsorship to remain independent from commercial interests [28] or a channel whose programming is primarily funded by advertising [29]. This can be explained by the broadcasts on the non-commercial channel showing video segments that featured examples of alcohol marketing instead of commercial advertising breaks. There was also no difference for the volume of alcohol marketing for broadcasts in the afternoon (e.g., 14:00 or 17:00 GMT) compared to the evening. This is important as more young people may have watched the games with an earlier start time, with anecdotal evidence suggesting that some schools allowed students to watch high-profile matches during school hours [30] or suggestions that some employers screened matches to staff to improve working morale [31]. That the volume of marketing was consistently high across the tournament is therefore consistent with suggested concerns that sport sponsorship may help to normalise alcohol consumption [32,33].

A unique characteristic of this study is that it examined references at an international football tournament held in France, a country with restrictive alcohol marketing regulations. In the early stages of coding it was identified that broadcast footage of each match was identical across the three countries. We confirmed this by obtaining recordings from matches broadcast in both France and Ireland and conducting a detailed comparison of in-match footage from broadcasts in both France (England versus Russia) and Ireland (Ireland versus Sweden) with the same matches as broadcast in the UK. This was consistent with press coverage explaining that international broadcasters joined the “world feed” supplied by UEFA’s International Broadcast Centre and overlaid their own commentary [34,35,36]. Therefore, in-play footage for all matches was the same in all three countries whereas the out-of-play sections (e.g., pre-match and post-match studio discussions) were different. The results, however, indicate that alcohol companies were able to circumvent the regulations to ensure alcohol marketing references were present at the tournament, despite the Loi Évin prohibiting marketing through sport sponsorship [11,12]. Instead marketing references were frequently reported in high-profile locations throughout broadcasts, in particular on electronic pitch side advertising during the match when the audience size is likely to be at its peak. Despite frequent presence, however, almost all marketing references were indirect in nature. In such references, no brand name or logo appeared but the brand was still identifiable from other signifiers such as phrases from the brand slogan, colours and typeface used. This suggests that the Loi Évin had an impact on the presence of alcohol marketing at the tournament, by ensuring brand names were for the most part not shown, but still represents a circumvention of the regulations as these references were paid-for media as part of the sponsorship rights afforded to Carlsberg. 

Although it was indirect references that appeared most frequently and in highly visible locations, the results also show that subtle and consumer-endorsed marketing practices were used to directly promote alcohol brands during the tournament, particularly through merchandise or products in the stadium crowds. Such references present unique challenges to enforcing marketing regulations because, although they are intentional marketing, their appearance in broadcasts is underpinned by individual consumer decisions. For example, during the tournament Carlsberg’s Icelandic distributor reportedly arranged to sell customized Iceland national team shirts with the brand’s logo prominent [37,38]. These branded shirts then featured prominently on supporters in the crowds during broadcasts of high-profile matches, including those of particular interest to UK audiences (England versus Iceland) and audiences in the host country (France versus Iceland). Other examples included branded beer cups (as a 0.5% version of Carlsberg was available in the stadiums), replica football jerseys for football clubs which have an alcohol sponsor (e.g., an Everton shirt with Chang beer), and Carlsberg-branded wigs with hair coloured to match nations’ flags. 

The Loi Évin has had previous success in restricting alcohol marketing in sports tournaments, including the 1998 FIFA World Cup [39] and 2007 Rugby World Cup [40]. Despite these previous tough stances, there is recent evidence of indirect alcohol marketing appearing again at sporting events in France. This has been achieved through “alibi marketing”, a practice that emerged in the 1990s to promote tobacco products within “dark markets” where tobacco advertising was restricted [41]. Alibi marketing involves distilling a brand identity into its key components (e.g., phrases from a slogan) and using these in place of a conventional logo or name. This strategy has since been adopted by alcohol marketers, with examples including Heineken’s sponsorship of the European Rugby Champions Cup, which was rebranded as the “H Cup” in France [42], and pitch side advertising during UEFA Champions League games, where Heineken’s usual branded pitch border advertising was replaced in French matches with an “Enjoy Responsibly” message which featured their red star logo on a green background [43]. Our study therefore provides further evidence of alibi marketing being used in France to promote alcohol. This offers a potential explanation why recent cross-sectional survey research reported that over a third of young people in France recalled seeing alcohol marketing at sporting events and concerts at least once in the past month [11]. Market research conducted by Carlsberg has confirmed that viewers made the connection between the alibi marketing displayed at the UEFA EURO 2016 tournament and the beer brand. Carlsberg reported that viewer recall of their brand for the tournament was 50 per cent. This was reportedly higher than other sponsors Coca-Cola and McDonald’s and that only McDonald’s “I’m lovin’ it” slogan achieved higher viewer recall than Carlsberg’s “Probably, the best in the world” [44].

There are two potential explanations for the presence of alcohol marketing at UEFA EURO 2016. The first is that the Loi Évin is not currently equipped to address alibi marketing. The basis of the law, however, is that the forms of marketing which are allowed are explicitly specified (with sport sponsorship and TV advertising explicitly prohibited), and any form of marketing that is not explicitly stated in the law is still considered to be prohibited [12]. Therefore, the fact that alibi marketing is not specified in the Loi Évin did not provide a mandate for it to be used at UEFA EURO 2016 and cannot explain the high presence of marketing we found. 

The second explanation is that the law is not being correctly enforced or adhered to by marketers. Consistent with this suggestion, we observed that even when marketing did appear, most examples did not comply with other Loi Évin regulations which state that marketing must be restricted to factual information about the product and must contain health warnings. “Probably the best in the world” is not simple factual information about the Carlsberg product and, apart from during the commercial breaks which fell under UK legislation, no health warnings accompanied the marketing (e.g., on pitchside advertising). Previous research suggests that it is not unusual for alcohol sponsorship of sport to not comply with existing national policies during intentional tournaments. For example, an analysis of broadcasts of the 2014 FIFA World Cup in eight countries found that advertisements during games contained content which violated self-regulatory codes [45]. Experiences at other international football tournaments, including the 2014 FIFA World Cup in Brazil and the scheduled 2018 FIFA World Cup in Russia suggest that existing policies can be diluted to coincide with major tournaments [46,47,48]. 

The alcohol industry has defended their marketing activity at UEFA EURO 2016. Carlsberg, the brand whose slogan appeared in most of the marketing references recorded, have stated that they comply with the legal requirements of all countries in which they operate and strictly applied the Loi Évin law by not advertising their beer brands and did not link their partnership to alcohol [49,50]. This defence, however, has been contested by the Association Nationale de Prevention en Alcoologie et Addictologie, an organization who address alcohol-related harm in France, who are intending to pursue legal action against the brands whose marketing appeared at the tournament [51]. 

### Limitations and Future Directions

The coding framework we used showed high inter-rater reliability, which indicates that the results are likely to be reliable. Nevertheless, the study does have three limitations. First, this study expanded on the coding frameworks used in previous studies to explore where, what and how alcohol marketing references were made. Although these amendments provide greater detail about marketing (for example, regarding the number of identical references that appeared at any one time and the duration they were on the screen), the change in coding procedure means that the findings cannot be directly compared to the frequency of references in the previous tournament, UEFA EURO 2012 [16]. As the previous tournament was held in Poland and Ukraine, where the regulations on alcohol marketing are not as strong as in France [10], use of an identical coding protocol would have provided a quasi-experimental means of evaluating to what extent, if at all, the Loi Évin reduced the presence of marketing. Future studies should consider using standardised coding procedures to evaluate marketing across sporting tournaments to enable objective comparisons between countries with different regulatory approaches.

Second, this study only considered UK broadcasts of UEFA EURO 2016. While the in-play footage was identical in all countries, as all international broadcasters joined a central UEFA feed before the match kicked off [33,34,35], almost 40% of the references appeared in out-of-play segments (e.g., pre-match, half-time and post-match). As regional broadcasters have greater autonomy over these segments, the results cannot be generalised to other countries. Future research should therefore focus on broadcasts of the same matches in different countries, to understand the impact of country-specific regulatory approaches (e.g., commercial advertising breaks are not permitted in France, whereas they were in the UK).

Third, as this study only considered an international football tournament, the findings may not be generalizable to other forms of televised football. For example, sponsor logos were not permitted on the players’ match shirts during the tournament (although “Carlsberg” logos were observed on replica Icelandic national team shirts worn by supporters in the crowd). As shirt sponsorship is permitted in many club-level competitions, such as England’s Premier League, it is possible that the frequency of marketing references may be greater in broadcasts of these matches [23]. Future research should therefore explore how the frequency, volume and type of alcohol marketing references differs between international and national-level sporting events and the potential implications this has for exposure.

## 5. Conclusions

This study adds to a growing body of evidence confirming that international football tournaments provide a high profile platform to market alcohol. The results help to extend previous research by exploring in detail the location, format and nature of alcohol references during broadcasts. The majority of marketing references were indirect in nature, demonstrating a growing use of alibi marketing to circumvent marketing restrictions. To ensure the spirit of the Loi Évin regulations are achieved, stricter enforcement may be required to limit exposure to alcohol marketing references, particularly for young people.

## Figures and Tables

**Table 1:** Characteristics of the eighteen matches as broadcast on UK television.

Date	Teams	Time (GMT)	Channel	Length (min)
*Group stage (n = 9)*				
Friday 10 June	France vs. Romania	20:00	STV	184
Saturday 11 June	England vs. Russia	20:00	ITV	208
Monday 13 June	Republic of Ireland vs. Sweden	17:00	BBC One	155
Wednesday 15 June	France vs. Albania	20:00	STV	167
Thursday 16 June	England vs. Wales	14:00	BBC One	179
Saturday 18 June	Republic of Ireland vs. Belgium	14:00	ITV	211
Sunday 19 June	France vs. Switzerland	20:00	BBC One	154
Monday 20 June	England vs. Slovakia	20:00	ITV	193
Wednesday 22 June	Republic of Ireland vs. Italy	20:00	STV	180
*Round of 16 (n = 3)*				
Sunday 26 June	France vs. Republic of Ireland	14:00	STV	182
Monday 27 June	Italy vs. Spain	17:00	BBC One	153
Monday 27 June	England vs. Iceland	20:00	STV	212
*Quarter-Finals (n = 3)*				
Thursday 30 June	Poland vs. Portugal	20:00	STV	224
Friday 1 July	Wales vs. Belgium	20:00	BBC One	160
Sunday 3 July	France vs. Iceland	20:00	STV	182
*Semi-Finals (n = 2)*				
Wednesday 6 July	Portugal vs. Wales	20:00	ITV	194
Thursday 7 July	Germany v France	20:00	BBC One	155
*Final (n = 1)*				
Sunday 10 July	Portugal vs. France	20:00	BBC One	243

**Table 2:** Descriptive statistics and independent samples *t*-tests comparing alcohol marketing references across different types of broadcast.

Variable	*n*	Mean	SD	*t*	*p*
*Overall*	18	122.94	42.87	-	-
*National focus*				−0.05	n.s.
Matches involving England	4	120.29	46.35		
Matches involving other teams	14	132.25	30.92		
*Broadcast channel*				−0.56	n.s.
BBC	7	115.71	49.34		
ITV/STV	11	127.55	40.04		
*Day of match*				0.31	n.s.
Weekday (Monday–Thursday)	10	125.80	35.86		
Weekend (Friday–Sunday)	8	119.38	52.75		
*Time of kick off*				0.08	n.s
Afternoon (14:00 and 17:00 GMT)	5	124.20	22.11		
Evening (20:00 GMT)	13	122.46	49.39		

n.s.: Non-significant.

**Table 3:** Location of the alcohol references.

Reference Characteristic	*n*	%
*Broadcast segment*		
Pre-match	374	16.9
First half	567	25.6
Half-time	165	7.5
Second half	701	31.6
Extra time	86	3.9
Breaks in extra time	27	1.2
Penalties	7	0.3
Post-match	249	11.3
Commercial break	37	1.7
Total in play	1361	61.5
Total out of play	852	38.5
*Location in broadcast*		
Pitch border	1724	77.9
Video Segment	326	14.7
Crowd	49	2.2
Interview area	44	2.0
Commercial break	37	1.7
Sponsorship lead in	19	0.9
Stadium interior	13	0.6
TV studio	1	<0.01

**Table 4:** Format and content of alcohol references.

Reference Characteristics	*n*	%
*Format of reference*		
Electronic pitch side (all)	838	37.9
Electronic pitch side (part)	797	36.0
Static advertising	445	20.1
Branded merchandise	56	2.5
Commercial break	37	1.7
Sponsorship lead-in	19	0.9
Product or packaging	17	0.8
Other	4	0.1
*Type of reference*		
Visual only	2157	97.5
Verbal and visual	56	2.5
*Nature of reference*		
Indirect reference	2006	90.6
Direct reference	207	9.4
*Alcohol brands featured*		
Carlsberg	2129	96.2
Other	84	3.8

## Appendix A

**Table A1:** Variables and definitions measured in the frequency analysis codebook.

Variable	Definition(s)
*Time of reference*	Recorded in hours, minutes and seconds, using the start of the broadcast as the 00:00:00 reference.
*Broadcast segment*	Broadcast segment was recorded as one of: (1) Pre-match—from beginning of broadcast until the first half begins; (2) First half—as indicated by match referee; (3) Half-time—from end of first half until start of second half, as indicated by match referee; (4) Second half, as indicated by match referee, including injury time; (5) Extra-time—any open play required after the conclusion of the second half in order to settle the match; (6) Breaks in extra-time—coverage occurring during breaks in extra time, as indicated by the match referee; (7) Penalties—after the end of extra-time and until the match result has been settled; (8) Post-match—content after the referee has indicated the end of the match until end of the programme; and (9) Commercial breaks during the broadcasted programme, not including those immediately before or after.
*Location*	One of: (1) Stadium crowd; (2) Field of play; (3) Interview area; (4) Pitch border to start of the stadium crowd; (5) On-screen graphics; (6) Short video introductions featuring the tournament sponsors which begin or end a segment of the broadcast; (7) TV studio; (8) Stadium interior—any temporary of fixed features in the stadium not covered in other categories (e.g., sponsorship boards on upper tiers); (9) Pre-recorded video segments (e.g., highlights of other matches); (10) Off-screen verbal references (e.g., match commentator); and (10) other.
*Type*	Recorded as visual (e.g., advertising board), verbal (e.g., commentator reference) or both (e.g., commercial advert with voiceover).
*Format*	One of: (1) Static advertising fixed in location (e.g., logos on interview boards); (2) Electronic all—instances of electronic/dynamic advertising which covers all the available pitch side boards; (3) Electronic part—instances of electronic/dynamic advertising which only covers part of available pitch side boards; (4) Product or packaging; (5) Verbal reference; (6) Branded merchandise—any item other than the actual product which display a brand logo, slogan or image; (7) Integrated on-screen graphic—such as logos used to transition between broadcast sections (e.g., into match replays, opening credits); (8) Short video introductions featuring the tournament sponsors which begin or end a segment of the broadcast; and (9) Other.
*Duration*	Total duration the reference lasted for (seconds).
*Number of identical references visible at same time*	The maximum total number of identical references visible on screen *at any one point*, not the maximum number visible across the total duration of the reference.
***Nature of the alcohol brand reference***	One of: (1) Direct brand reference—such as brand names or logos; (2) Indirect brand reference—although a brand name did not appear, the brand was still identifiable from other signifiers such as phrases from the brand slogan, colour, and typeface. Generic references to alcohol where there was no obvious brand link (such as commentators mentioning that fans or players might “enjoy a beer”) were excluded.
*Alcohol brand featured*	If a direct or indirect brand reference, then the name of the alcohol brand depicted was recorded.

## Appendix B

**Table A2:** Summary of inter-rater reliability agreement.

Variables	NC	RP	% Agreement
	*n*	*n*	
Broadcast segment			
*Pre-match*	23	21	91%
*1st half*	37	37	100%
*Half-time*	15	15	100%
*2nd half*	50	50	100%
*Post-match*	6	5	83%
Average agreement for variable			95%
Reference type			
*Visual*	131	128	98%
Location			
*Pitch border*	107	105	98%
*Video segment*	21	20	95%
*Stadium interior [Temporary]*	3	3	100%
Average agreement for variable			98%
Format			
*Electronic advertising (all)*	66	67	99%
*Electronic advertising (part)*	48	43	90%
*Static advertising*	17	18	94%
Average agreement for variable			94%
Content of reference			
*Indirect brand reference*	124	121	98%
*Direct brand reference*	7	7	100%
Average agreement for variable			99%
Brand			
*Carlsberg*	124	121	98%
*Other (Metaxa brandy)*	7	7	100%
Average agreement for variable			99%

**Details:** Nathan Critchlow versus Richard I Purves; Semi-Final 2 (SEM02)—France versus Germany.

## Appendix C

**Table A3:** Frequency of all alcohol brands present in UK broadcasts of UEFA EURO 2016.

Brand Name	Number of References
Carlsberg	2129
Budweiser	17
Metaxa	14
Fosters	6
Carling	6
Brain’s Brewery	4
Vin Italia	4
Strongbow	4
Kronenbourg	4
Chang	3
Captain Morgan’s	3
Jim Bean	3
Amstel	2
Belhaven	2
Unsure/Multiple brands	2
Guinness	1
Holsten Pils	1
Worthington’s	1
Chatena La Garde	1
Coors	1
Jupiler	1
Sagres	1
Thatcher’s Cider	1
Blossom Hill	1
Magner’s	1

## References

[1] Hastings G., Brooks O., Stead M., Angus K., Anker T., Farrell T. (2010). “They’ll Drink Bucketloads of the Stuff”: An Analysis of Internal Alcohol Industry Documents.

[2] Purves R.I., Stead M., Eadie D. (2014). “What are you Meant to do When you See it Everywhere?” Young People, Alcohol Packaging and Digital Media.

[3] Casswell S., Zhang J.F. (1998). Impact of liking for advertising and brand allegiance on alcohol-related aggression. Addiction.

[4] McClure A.C., Stoolmiller M., Tanski S.E., Engels R.C., Sargent J.D. (2013). Alcohol marketing receptivity, marketing-specific cognitions, and underage binge drinking. Alcohol. Clin. Exp. Res..

[5] Tanski S., McClure A.C., Jernigan D.H., Sargent S.D. (2011). Alcohol brand preference and binge drinking among adolescents. Arch. Pediatr. Adolesc. Med..

[6] Brown K. (2016). Association between alcohol sports sponsorship and consumption: A systematic review. Alcohol Alcohol..

[7] Burton R., Henn C., Lavoie D., O’Connor R., Perkins C., Sweeney K., Greaves F., Ferguson B., Beynon C., Belloni A. (2016). A rapid evidence review of the effectiveness and cost-effectiveness of alcohol control policies: An English perspective. Lancet.

[8] Jernigan D.H., Noel J., Landon J., Thornton N. (2017). Alcohol marketing and youth alcohol consumption: A systematic review of longitudinal studies since 2008. Addiction.

[9] Leyshon M., Sakhuja R. (2013). A Losing Bet? Alcohol and Gambling: Investigating Parallels and Shared Solutions.

[10] World Health Organization (2014). WHO Global Status Report on Alcohol and Health 2014.

[11] Gallopel-Morvan K., Spilka S., Mutatayi C., Rigaud A., Lecas F., Beck F. (2017). France’s Évin Law on the control of alcohol advertising: content, effectiveness and limitations. Addiction.

[12] Lecas F. How Loi Evin is working and its challenges. Proceedings of the 7th European Alcohol Policy Conference.

[13] Robert J., Flowers R., Swan E. (2015). The Loi Evin: A Pedagogical Experiment in Responsible Drinking. Food Pedagogies.

[14] Kantar Media (2014). 2014 FIFA World Cup Brazil, Television Audience Report.

[15] UEFA Hisense signs as UEFA EURO 2016 Global Sponsor. http://www.uefa.org/mediaservices/mediareleases/newsid=2323656.html.

[16] Adams J., Coleman J., White M. (2014). Alcohol marketing in televised international football: Frequency analysis. BMC Public Health.

[17] Alcohol Concern (2014). Alcohol Marketing at the FIFA World Cup 2014: A Frequency Analysis.

[18] UEFA Impressive Global Broadcast Coverage of UEFA EURO 2016. http://www.uefa.org/mediaservices/newsid=2373525.html.

[19] Carlsberg Carlsberg’s UEFA EURO 2012 Campaign Sets New Standards. http://www.carlsberggroup.com/media/news/Pages/PR11_Euro_end_03072012.aspx.

[20] Alcohol Health Alliance (2013). Health First: An Evidence Based Alcohol Strategy for the UK.

[21] Babor T.F., Caetano R., Casswell S., Edwards G., Giesbrecht N., Graham K., Grube J.W., Hill L., Holder H., Homel R. (2010). Alcohol: No Ordinary Commodity: Research and Public Policy.

[22] Hastings G., Sheron N. (2011). Alcohol marketing to children. BMJ.

[23] Graham A., Adams J. (2014). Alcohol Marketing in Televised English Professional Football: A Frequency Analysis. Alcohol Alcohol..

[24] Alcohol Action Ireland (2014). Submission to the Working Group on Regulating Sponsorship by Alcohol Companies of Major Sporting Events.

[25] D’Arcy C. (2015). Alcohol Bill to undermine Rugby World Cup bid—Drinks lobby. The Irish Times.

[26] Plunkett J. (2016). Andy Murray’s Wimbledon nets 13.3 m viewers—But more see Euro 2016 final. The Guardian.

[27] Stemler S., Tsai J., Osborne J. (2008). Best practices in estimating interrater reliability. Best Practices in Quantitative Methods.

[28] British Broadcasting Corporation Inside the BBC: Advertising. http://www.bbc.co.uk/corporate2/insidethebbc/howwework/policiesandguidelines/advertising.html.

[29] ITV About ITV. http://www.itvplc.com/about/what-we-do.

[30] Evans G. (2016). It is up to head teachers whether pupils and staff will be able to watch Wales’ EURO 2016 Showdown with England. Wales Online.

[31] Bullen J. (2016). England vs. Wales: Firms told to show Euro 2016 shown to ‘motivate staff’. The Evening Standard.

[32] Campbell D. (2014). Ban alcohol firms from sponsoring sports clubs and events, doctors urge. The Guardian.

[33] Douglas A. (2016). Let’s not normalise alcohol sport. Edinburgh News.

[34] Nield D. (2016). Broadcast tech explainer—How Euro 2016 is beamed into your eyes and ears. The Gadget Website.

[35] Roder J. World Feed Football Commentary: What Is It?. http://johnroder.com/blog/world-feed-football-commentary.-what-is-it.

[36] Temperton J. (2016). Fibre and plywood: This is how EURO 2016 is broadcast to the world. Wired Sport.

[37] Glenndinning M. (2016). Five things we learnt from Carlsberg’s Campaign. Sports Business.

[38] McCarthy J. (2016). How Carlsberg made you want beer at Euro 2016 despite being legally forbidden from using its branding. The Drum.

[39] Hare G., Dauncey H., Hare G. (1999). Buying and selling the world cup. France and the 1998 World Cup: The National Impact of a World Sporting Event.

[40] Palmer C. (2015). Rethinking Drinking and Sport: New Approaches to Sport and Alcohol.

[41] Grant-Braham B., Britton J. (2012). Motor racing, tobacco company sponsorship, barcodes and alibi marketing. Tob. Control.

[42] Jones C.R. (2016). Sport and Alcohol: An Ethical Perspective.

[43] Heineken Heineken ‘Champions the Match’ in new UEFA Champions League Campaign. http://www.theheinekencompany.com/media/media-releases/media-releases/2015/10/heineken-champions-the-match-in-new-uefa-champions-league-campaign.

[44] Jancey N.V. EURO 2016 Cyber Security in Practice. https://www.pwc.dk/da/arrangementer/2016/cybercrimekonferencen16carlsberg.pdf.

[45] Noel J.K., Babor T.F., Robaina K., Feulner M., Vendrame A., Monteiro M. (2017). Alcohol marketing in the Americas and Spain during the 2014 FIFA World Cup Tournament. Addiction.

[46] European Centre for Monitoring Alcohol Marketing Due to FIFA World Cup: Restrictions on Advertising Beer in Russia Eased?. http://eucam.info/2016/12/20/due-to-fifa-world-cup-restrictions-on-advertising-beer-in-russia-eased/.

[47] Gornall J. (2014). World Cup 2014: festival of football or alcohol? Whichever country hoists aloft the World Cup trophy on 13 July, the real winner will be the alcohol industry. BMJ.

[48] Nicholls J., Kolind T., Thom B., Hunt G. (2016). Buying and selling the world cup. Handbook of Drug & Alcohol Studies: Social Science Approaches.

[49] Flynn V. (2017). Brewer scores past the French: Ease with which Carlsberg slips past Euro 2016 alcohol advertising ban poses questions for Irish bill. The Sunday Times.

[50] Guernsey Press (2017). Alcohol companies targeted ‘global audience’ with Euro 2016 campaign: Alcohol companies bent the rules by advertising during the Uefa Euro 2016 tournament, placing children’s health at risk, according to a report. Guernsey Press.

[51] Association Nationale de Prevention en Alcoologie et Addictologie Game of dupes? Report Highlights Alcohol Industry Practices to Bypass Rules on Advertising during Euro 2016 [Press Release]. http://www.anpaa.asso.fr/presse/espace-presse/890-jeu-dupes-rapport-met-en-lumiere-pratiques-industrie-alcool-contourner-regles-publicite-euro-2016.

